# Immunological and viral features in patients with overactive bladder indicate an early stage of myelopathy

**DOI:** 10.1186/1742-4690-8-S1-A116

**Published:** 2011-06-06

**Authors:** Silvane B Santos, Paulo Oliveira, Matheus Tannus, Tania Luna, Anselmo Souza, Thais Delavechia, Márcia Nascimento, Glória Orge, Isadora Siqueira, Davi Tanajura, André Luiz Muniz, Marshall J Glesby, Edgar M Carvalho

**Affiliations:** 1 Serviço de Imunologia, Complexo Hospitalar Professor Edgard Santos, Universidade Federal da Bahia, Salvador, Bahia, Brazil; 2 Departamento de Ciências Biológicas, Universidade Estadual de Feira de Santana, Bahia, Brazil; 3 Instituto Nacional de Ciência e Tecnologia de Doenças Tropicais (CNPq/MCT), Salvador, Bahia, Brazil; 4 Weill Cornell Medical College, New York, NY, USA

## Background

The majority of Human T cell lymphotropic virus type 1 (HTLV-1) infected subjects are considered as HTLV-1 carriers but a high frequency of urinary manifestations of overactive bladder has been documented in these individuals. The aim of this study was to determine if viral and immunological factors that are associated with development of HTLV-1-associated myelopathy/tropical spastic paraparesis (HAM/TSP) are also observed in HTLV-1 patients with overactive bladder.

## Materials and methods

This was a cross-sectional study comparing immunological responses and proviral load in 3 groups of HTLV-1 infected subjects: HTLV-1 carriers (n=45), HTLV-1 overactive bladder (n=45) by International Continence Society (ICS) criteria and HAM/TSP patients (n=45) according WHO guidelines.

## Results

We demonstrated that cells from HTLV-1 overactive bladder produce higher spontaneous levels of IFN-γ, TNF-α and IL-17 than carriers and similar levels of TNF-α and IL-17 to HAM/TSP. Proviral load was higher in HTLV-1 overactive bladder and HAM/TSP than carriers and correlated positively with production of proinflammatory cytokines. In contrast to HAM/TSP, HTLV-1 overactive bladder had serum levels of Th1 chemokines similar to carriers and exogenous addition of regulatory cytokines (IL-10 and TGF-β) decreased IFN-γ production in cell cultures from HTLV-1 overactive bladder.

## Conclusions

We conclude that HTLV-1 overactive bladder patients have some immunological features and proviral load profiles in common with HAM/TSP. However as they are still able to down modulate the inflammatory response and the recruitment of activated T cells to the central nervous system is not enhanced, they present an oligosymptomatic form of HAM/TSP.

